# Scientific Basis for the Potential Use of Melatonin in Bone Diseases: Osteoporosis and Adolescent Idiopathic Scoliosis

**DOI:** 10.4061/2010/830231

**Published:** 2010-06-01

**Authors:** E. J. Sánchez-Barceló, M. D. Mediavilla, D. X. Tan, R. J. Reiter

**Affiliations:** ^1^Department of Physiology & Pharmacology, School of Medicine, University of Cantabria, 39011 Santander, Spain; ^2^Department of Cellular & Structural Biology, University of Texas Health Science Center, San Antonio, TX 78229, USA

## Abstract

The objective of this paper was to analyze the data supporting the possible role of melatonin on bone metabolism and its repercussion in the etiology and treatment of bone pathologies such as the osteoporosis and the adolescent idiopathic scoliosis (AIS). Melatonin may prevent bone degradation and promote bone formation through mechanisms involving both melatonin receptor-mediated and receptor-independent actions. The three principal mechanisms of melatonin effects on bone function could be: (a) the promotion of the osteoblast differentiation and activity; (b) an increase in the osteoprotegerin expression by osteoblasts, thereby preventing the differentiation of osteoclasts; (c) scavenging of free radicals generated by osteoclast activity and responsible for bone resorption. A variety of in vitro and in vivo experimental studies, although with some controversial results, point toward a possible role of melatonin deficits in the etiology of osteoporosis and AIS and open a new field related to the possible therapeutic use of melatonin in these bone diseases.

## 1. Introduction

Bones are structures under a continuous process of remodelating by the coupled activity of cells with resorptive functions (osteoclasts) and cells responsible for the formation of new bone (osteoblasts). The balance between the activities of both cell types is under the control of systemic hormones including parathyroid hormone (PTH), estradiol (E2), and growth hormone as well as of cytokines and growth factors produced in the bone marrow [1]. A major disease of bone, osteoporosis, has been defined as “a systemic disease characterized by low bone mass and micro architectural deterioration of bone tissue, with consequent increase in bone fragility and susceptibility to fracture”. This is a disease predominantly associated with aging, with a special prevalence among women [2]. Adolescent idiopathic scoliosis (AIS) is the most common type of scoliosis and also is more prevalent among females, especially during prepuberal and puberal growth, when bone acquisition is highest [3]. 

Melatonin is an indoleamine secreted primarily by the pineal gland but also synthesized in other organs such as retina, gastrointestinal tract, and bone marrow. Melatonin plays a regulatory role in many physiological processes including bone physiology [4–10]. Nocturnal plasma melatonin levels significantly decline after the age of 50 in both genders [11, 12]. Since the time course of the reduction of melatonin production and the progression of bone deterioration run in parallel, the possible role of melatonin in osteoporosis has been considered worthy of study. Regarding AIS, the fact that experimental pinealectomy in different animal models [13–22] results in scoliosis which closely resembles the human pathology opened a new field of research on the role of melatonin in the AIS.

The objectives of the current paper are (a) to review the data supporting the possible role of the age-dependent decrease of melatonin in the development of osteoporosis and the therapeutic value of melatonin as a treatment for this disease, and (b) to analyze the evidence related to the role of melatonin in the etiology and treatment of the AIS. Before doing so, we will describe the effects of melatonin on bone physiology, as the basis to understand the participation of this indoleamine in bone pathology.

## 2. Melatonin and Bone Physiology

The effects of melatonin on bone physiology were reviewed in an excellent article by Cardinali et al. [4]. The possible influence of melatonin on bone metabolism was repeatedly proposed by different authors during the last four decades [23–27]. These proposals were made on the basis of evidence for the pineal control of the secretion of parathyroid hormone and calcitonin, demonstrable by the ultrastructural and functional changes observed in parathyroid glands after pinealectomy. The earliest experiments examined the influence of the pineal on calcemia. It was observed, for example, that the inhibition of melatonin synthesis by exposure of newborn rats to white fluorescent light reduced the concentration of calcium in the serum [28]. This effect was prevented by exogenous melatonin administration. Light-induced hypocalcemia may result from augmented calcium uptake by bone when melatonin levels are reduced after inhibition of its synthesis by light [28]. Likewise, when melatonin secretion was inhibited in rats by the administration of *β*-adrenoceptor blockers, serum concentrations of calcium dropped [29] an effect which was also prevented by the administration of melatonin. The conclusion from these experiments is that suppression of melatonin causes hypocalcemia and additionally suggesting that melatonin would normally upregulate the blood levels of calcium.

More recently, Ostrowska et al. [30] re-examined, in male rats, the effects of the exposure to different lighting conditions not only on calcemia but also on bone physiology. They did this by evaluating the influence of alterations in the light:dark cycle on biochemical markers of bone metabolism (serum alkaline phosphatase, concentration of carboxyterminal propeptide of type I procollagen, cross-linked carboxyterminal telopeptide of type I collagen, inorganic phosphorus, urinary excretion of hydroxyproline and calcium). They reported that short days (LD 0.5:23.5 h) had a stimulatory effect on the level of these markers, while exposure to long days (LD 23.5:0.5 h) was inhibitory. Anomalies in daily oscillations of these markers with a negative correlation with the changes in endogenous melatonin concentrations and a positive correlation with daily fluctuations of IGF-I and triiodothyronine (T_3_) were also described. These results led the authors to conclude that lighting conditions influence bone metabolism in rats, and that melatonin likely plays an important role in these photoperiodic effects. Secondary changes in daily IGF-I and T_3_ oscillations, caused by short- and long-day conditions, also result in altered rhythmicity of daily bone resorption [30]. This experiment demonstrated the possible influence of melatonin on bone metabolism but not its concrete effects on bone formation and resorption. However, positive effects of melatonin on osteoblastic activity were deduced from the increases in the formation of cortical bone in mice treated with intraperitoneal injections of the indoleamine [31]. 

One interesting finding potentially related to melatonin and bone health is the demonstration of high concentrations of melatonin in bone marrow cells from mice and humans [32, 33], with the concentrations being approximately twice as high as nighttime levels in peripheral blood [32]. The cells in question contain aryl-alkyl-N-acetyltransferase activity and express the mRNA encoding hydroxyindole-O-methyltransferase, indicating the ability of the cells to synthesize melatonin *de novo* [33]. Moreover, human osteoblasts express MT1 melatonin receptors, and its expression level decreases with the age of the host [31]. The presence of melatonin in bone marrow may be protective against oxidative damage in the proliferating hematopoietic cells or involved in bone development through osteoblast differentiation [34, 35]. 

A variety of in vitro studies support the hypothesis of stimulatory effects of melatonin on both osteoblast differentiation and activity. Preosteoblasts cultured in the presence of melatonin underwent early cell differentiation and a major expression of bone marker proteins compared to control cells incubated without melatonin [36]. These effects are prevented by the melatonin receptor antagonist luzindole [36]. The age-related decrease of melatonin production could shift the bone marrow cells differentiation from osteoblastic differentiation toward an adipocytic line of cell, which could explain the development of osteoporosis during aging [37]. Melatonin also promotes the osteogenic differentiation of bone marrow stem cells whereas it has negative effects on differentiation of adipose-derived stem cells [38, 39]. 

In cultures of human osteoblasts [31, 40], melatonin, at pharmacological doses (*μ*M range), (a) stimulates the proliferation and alkaline phosphatase activity of these cells; (b) promotes the expression of type I collagen, osteopontin, bone sialoprotein, and osteocalcin; (c) stimulates the formation of mineralized matrix. The signaling mechanisms mediating the melatonin actions on osteoblasts are still unknown although the role of the MAPK pathway seems relevant [35].

The activity of osteoclasts is under the control of paracrine factors produced by the osteoblasts. PTH and 1,25-dihydroxycholecalcipherol stimulate the expression of an osteoclast differentiating factor (ODF) by the marrow stromal cells and osteoblasts. ODF binds to the receptor activator of nuclear factor-*κ*B (RANK) on the surface of the osteoclast activating bone resorption [4, 41]. In mouse osteoblasts, melatonin, at micromolar doses, decreases the expression of RANK mRNA and increases both the mRNA and protein levels of osteoprotegerin, a member of the super family of TNFR (tumor necrosis factor receptor) which inhibits the differentiation of osteoclasts by binding to ODF and preventing the binding of this factor to RANK [42]. Via this mechanism, melatonin could cause an inhibition of bone resorption and an increase in bone mass. 

One important component of the osteoclasts activity is the generation of free radicals which contribute to the process of bone degradation and resorption [43]. Melatonin, due to its ability to directly neutralize free radicals and to stimulate the activity of antioxidative enzymes [44, 45], may reduce osteoclastic activity.

**Figure 1:**
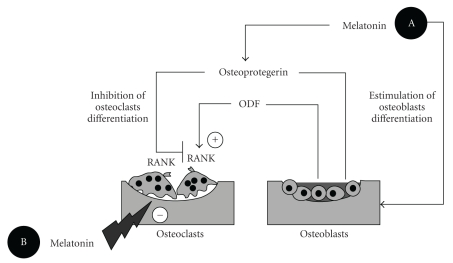
Effects of melatonin on bone metabolism. (a) Melatonin promotes the osteoblast proliferation and the synthesis osteoprotegerin, which inhibits the differentiation of osteoclasts by preventing the binding of ODF (osteoclast differentiation factor) to RANK on the differentiating osteoclasts. (b) Melatonin through its free radical scavenging properties impairs osteoclast activity on bone. Based on Cardinali et al. [4].

Figure 1 summarizes the principal mechanisms reviewed above related to melatonin's effects on bone function. These actions include: (a) the promotion of the osteoblastic differentiation, activity and expression of osteoprotegerin which prevents the differentiation of osteoclasts, and (b) scavenging of free radicals generated by osteoclastic activity and responsible for bone resorption.

There are, however, data contrary to the hypothesis of the effect of melatonin on bone-forming osteoblasts. Ostrowska et al. [46] found, in male Wistar rats, that high plasma concentrations of melatonin correlated with low levels of bone forming markers, and that pinealectomy elevated the levels of bone metabolism biomarkers and altered the phase and amplitude of its circadian rhythm. In another interesting study, Suzuki and Hattori [47] cultured osteoblasts in the presence of osteoclasts, analyzing the effects of melatonin on both, that is, osteoblastic and osteoclastic activity, by the changes on specific biomarkers in each cell type. They observed an inhibition by melatonin of the activity of both cell types. These authors emphasize the importance of the cell-to-cell interactions between osteoblasts and osteoclasts to understand their physiologic function as well as in the response to melatonin. Since melatonin inhibits both osteoblasts and osteoclasts, the final outcome of their effects could be the balance between the actions of these cellular elements. In postmenopausal women, bone resorption increases more than bone formation, thus resorption becomes the major determinant of bone mass [48]; in these cases, even therapies like melatonin, that may inhibit both osteoclasts and osteoblasts activity, should have positive effects on bone mass. 

An interesting question is the interaction of melatonin with estrogens at the level of the osteoblast. Estrogens have a positive impact on bone growth. Melatonin, according to the studies summarized above, has similar effects. Under many other circumstances, however, estrogens and melatonin usually have opposing effects. Thus, it is well known that estradiol modulates the function of the melatonin receptors in rat ovary [49] and Chinese hamster ovary cells [50], and that melatonin suppresses transcriptional activation of the ER*α* in MCF-7 cells by mechanisms involving calmodulin [51–53]. A study in goldfish scales showed that melatonin suppresses the activity of osteoblasts by downregulating the ER [47]; however, melatonin seems to enhance the effects of estradiol in the prevention of bone loss in ovariectomized rats [54]. The nature of the interactions of estradiol with melatonin on bone could be dependent of the estrogen concentration. In the above mentioned experiment, the prevention of the postovariectomy disruption of bone remodeling with pharmacological doses of melatonin required adequate concentrations of estradiol.

## 3. Melatonin and Osteoporosis

Osteoporosis is a prolonged structural deterioration of the skeletal system, usually associated with age, and with a major prevalence in women. Antiosteoporosis therapies include the use bisphosphonates, estrogen, and calcitonin to inhibit bone-resorbing osteoclasts preventing further bone breakdown. However, these therapies are insufficient in cases of individuals suffering from severe osteoporosis. Drugs that stimulate bone-forming osteoblasts (e.g., teriparatide) are expensive and with important associated side effects [5]. These facts and the above described effects of melatonin on bone physiology prompted studies on their possible utility as a complementary therapy for osteoporosis. Melatonin has been shown at the cell and tissue levels to promote osteogenesis and prevent bone deterioration in mammals [55], birds [56, 57], and fishes [21].

At present, no clinical trials have focused on the possible therapeutic value of melatonin in the treatment of osteoporosis. Some epidemiologic studies re-enforce the possible etiologic role of melatonin in osteoporosis. This is the case from a recent study of Feskanich et al. [58]. This group reported that in a sample of more than 38,000 postmenopausal women, compared with women who never worked night shifts, twenty or more years of night shift work significantly increased the risk of wrist and hip fractures over 8-year follow-up period. Night shift work causes disturbances in the patterns of melatonin secretion as well as severe circadian rhythm disruption [59]. 

Experimental studies carried out mostly in ovariectomized rats (as a model of menopause) suggest, in general, a protective role of melatonin in preventing bone degradation and promoting bone formation most probably through an action that involves melatonin receptors [4, 5]. Among these studies are those of Oktem et al. [60], suggesting that melatonin's prevention of osteoporosis could be related with its ability to inhibit inducible nitric oxide synthase (iNOS). iNOS plays a critical role in the pathogenesis of osteoporosis since it promotes the generation of nitric oxide, a free radical which contributes to bone resorption caused by estrogen depletion. By using the ovariectomized rat as a model, these authors demonstrated that melatonin treatment markedly reduced the expression of iNOS and the number of apoptotic cells in nucleus pulposus and epiphyseal cartilage of the spinal column, which increased after ovariectomy. Using the same animal model, Uslu et al. [61] described how trabecular thickness and trabecular area of vertebra and femur and cortical thickness of femur, which were significantly reduced after ovariectomy, increased after treatment with melatonin. Recently, Suzuki et al. [62, 63] developed a synthetic melatonin derivative, 1-benzyl-2,4,6-tribromomelatonin (bromomelatonin) which augmented the total bone mineral density of ovariectomized rats more efficiently than melatonin, suggesting its potential use in the treatment of osteoporosis.

## 4. Melatonin and Adolescent Idiopathic Scoliosis (AIS)

Although the etiology of the AIS is unclear, histomorphometric data on iliac crest biopsies and vertebrae of scoliosis patients showed an impaired function of both osteoblasts and osteoclasts [64, 65]. The persistent osteopenia in patients with AIS [64–66] and the effects of melatonin in bone metabolism stimulated several studies in animal models and humans related to the possible relationship between melatonin deficits and scoliosis [18, 19, 67–72]. 

**Figure 2:**
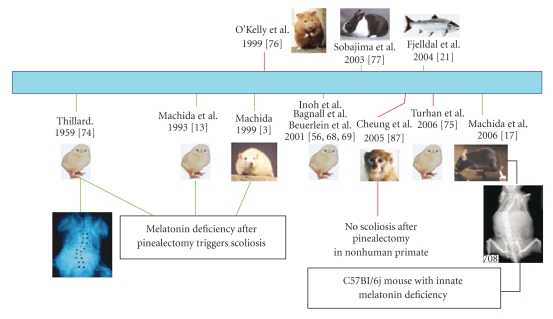
Summary of the experiments focused on the hypothesis involving a melatonin deficiency as the source for AIS. Effective (green lines) and noneffective (red lines) results are indicated. Modified from Moreau et al. [73].

The neuroendocrine hypothesis involving a melatonin deficiency as the source for AIS has generated great interest and controversy. This hypothesis, represented in Figure 2 (modified from Moreau et al. [73]), stems from the fact that experimental pinealectomy in the chicken [13, 14, 18–20, 74, 75], rats, and mice with genetic deficiency of melatonin forced into a bipedal mode of locomotion [16, 17, 22, 76], rabbits [77], and Atlantic salmon [21] results in scoliosis that closely resembles the AIS. Pinealectomy in chickens induces histomorphometric changes in the vertebral column. In particular, the loss of melatonin induces a scoliotic curvature and reduces mean weight and length of cervical vertebrae, possibly due to a reduction in the total number of osteocytes. These results were interpreted to mean that melatonin may act to enhance osteocyte proliferation in the cervical vertebrae [57]. 

In bipedal pinealectomized rats a reduction in melatonin, as a consequence of the pineal ablation, was found to cause scoliosis [14]. Recently, the possible role of calmodulin (CaM) as a mediator of the melatonin antiscoliosis effects has been proposed [78–80]. Melatonin is an inhibitor of calmodulin [81, 82] and, the loss of this inhibition, due to the lack of melatonin, could be the cause of scoliosis in these animal models. Since tamoxifen is working not only through estrogen receptor but act also as a CaM antagonist, pinealectomized chickens were treated with tamoxifen, and the incidence of scoliosis decreased, presumably due to CaM antagonism of this drug, although measures of CaM activity were not made. In a similar study, carried out on C57BL6 mice (which are genetically melatonin deficient), it was observed that they develop scoliosis when rendered bipedal; in these animals as well, tamoxifen improved the scoliosis deformities. In humans, Acaroglu et al. [78] compared the content of CaM and melatonin in muscle and platelets of scoliotic and healthy populations. The patients suffering with AIS had asymmetric distribution of CaM in the paraspinal muscles, with its concentration being higher at the convex side and lower at the concave curvatures of the spinal column, whereas neither platelet melatonin nor platelet CaM was found to be representative of the muscle protein values.

Not all data support the hypothesis of the reduction of melatonin as the cause of scoliosis. Melatonin therapy after pinealectomy in young chickens had no effect on the development or progression of scoliosis [83], and cutting of the pineal stalk of the chicken, without removal of the pineal gland, also resulted in scoliosis, whereas suppression of melatonin secretion by exposure of the chickens to constant light did not induce spinal curvature [84]. This suggests that the cause of the scoliosis is more related with the surgery than with the changes in melatonin secretion. Furthermore, although melatonin receptors are present in the spinal cord of the chicken, the changes detected in melatonin receptor binding after pinealectomy cannot explain why scoliosis develops in some chickens after pinealectomy, while it does not in others [85].

Bipedal ambulation in mammals is required, associated to low levels of melatonin, to generate scoliosis [22, 86]. The disturbance of equilibrium and other postural mechanisms secondary to a deficiency of melatonin may promote development of lordoscoliosis with vertebral rotation especially in the bipedal posture [86]. However, pinealectomized young rhesus monkeys (8–11 months old) do not develop scoliosis. This fact suggests that the possible etiologic factors producing idiopathic scoliosis in lower animals may be different from primate, and findings in birds and rodents cannot necessarily be extrapolated to human beings [87, 88]. Since monkeys in captivity, placed in cages that greatly restrict their mobility, spend most time in quadrupedal position, whether or not posture and gravity are determinants in the response to pinealectomy in terms of scoliosis is still unclear.

**Figure 3:**
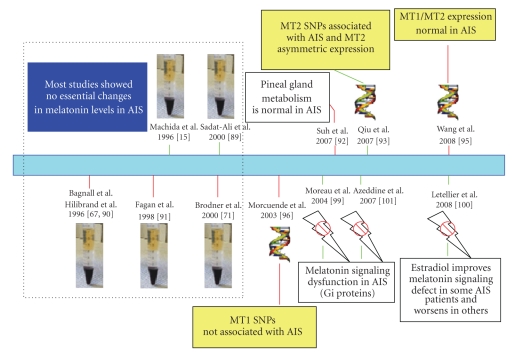
Summary of the main experimental approaches carried out in humans to clarify the role of melatonin on the AIS. Left, (doted rectangle), studies of changes in melatonin production. Yellow labels are the screening of polymorphisms in genes related with pineal function. White labels identify studies of possible changes in melatonin metabolism or response of target tissues. As in Figure 2, effective (green lines) and noneffective (red lines) results are indicated. Modified from Moreau et al. [73].

In humans, the question of the possible role of melatonin in scoliosis has been addressed using different analytical approaches (see Figure 3, modified from Moreau et al. [73]). One of these approaches was the detection of the possible changes in melatonin production in scoliotic patients. In this regard, Sadat-Ali et al. [89] found serum melatonin levels significantly lower in AIS patients than in healthy controls these results support the hypothesis that serum melatonin levels may contribute to the pathogenesis of idiopathic scoliosis. However, no significant difference between patients with AIS and controls regarding in serum concentration of melatonin or levels of urinary excretion of 6-sulfatoxy-melatonin was found by other authors; they concluded that a permanent melatonin deficiency is not a causative factor in the etiology of AIE in humans [67, 71, 90–92].

Genetic studies have screened AID and healthy patients looking for gene variants or single nuclear polymorphism in genes involved in the control of melatonin synthesis or in the expression of melatonin receptors. The screening of the MT2 receptor gene polymorphism in AIS patients and controls [93] suggests that this is a gene involved in the predisposition for AIS. However, the promoter polymorphism of the MT1 gene was not associated with the occurrence or curve severity of AIS, thus, indicating that MT1 gene may not be involved in the etiopathogenesis of AIS [94]. Polymorphisms of the arylalkylamine N-acetyltransferase (AANAT) gene were not associated with AIS whereas single nuclear polymorphism of tryptophan hydroxylase 1 gene (TPH1) seems closely related with the dysfunction of melatonin in AIS [95]. Other authors did not observe mutations in the coding region of the gene for human melatonin receptor in patients with familiar AIS [96].

A third category of studies have focused on the possible changes in melatonin receptors in AIS patients. The expression of MT2 melatonin receptors in bilateral paravertebral muscles in AIS and congenital scoliosis is asymmetric, being higher in muscles on concave side than that on convex side of the spinal column in AIS, but MT1 expression was not significantly different [97, 98]. These differences in the expression of melatonin receptors have been considered as secondary to the bilateral asymmetry due to force exerted on the scoliotic spine and not important in the pathogenesis of AIS [97, 98].

A different and interesting approach presented by Moreau et al. [99] could clarify the discrepancies regarding the role of melatonin in AIS. These authors consider that instead of changes in melatonin production or expression of melatonin receptors, the problem may be in the specific response of the osteoblast to melatonin in AIS patients. They demonstrated a melatonin signaling dysfunction occurring in osteoblasts isolated from AIS patients but not in similar cells isolated from healthy subjects. In most cells, melatonin inhibits the forskolin-stimulated adenylyl cyclase activity and decreases cAMP. In contrast, osteoblasts from patients with AIS showed a lack or a marked inhibition by melatonin of the forskolin-stimulated adenylyl cyclase activity [99]. The cause is an increased phosphorylation of serine residues affecting the activity of G-inhibitory proteins normally associated with melatonin surface receptors [99]. In response to estradiol, osteoblasts from a specific group of AIS patients treated with melatonin decreased the cAMP abnormally increased by the indoleamine [100]. From the findings of Moreau et al. [99], a preliminary molecular classification of AIS patients based on the cellular response to melatonin (changes in cAMP), has been proposed [101]. Recently, the same group [73] have developed the first blood test to detect children without symptoms who are at risk of developing scoliosis. This test is based on the cellular reaction to melatonin. The most recent clinical study on the relationship between melatonin and AIS has been a prospective analysis on the correlation of serum melatonin levels (monitored yearly for 3–6 years) and curve progression in 40 patient with moderate to severe AIS [102]. From 22 patients with normal melatonin levels (similar to healthy age-matched controls), 16 had stable scoliosis whereas 6 had progressive scoliosis. The 16 patients with low melatonin levels were treated with oral melatonin (3.0 mg 1.5–2.0 hour before the desired sleep time). Twelve of them developed stable scoliosis, whereas four continued to have progressive course. This is the first description of the therapeutic application of melatonin for this disease and suggests that melatonin supplementation could prevent the progression of the scoliosis, especially in mild cases. Obviously, more clinical trials are required to strengthen on the evidence regarding the benefits of melatonin and treatment for scoliosis.

## 5. Concluding Remarks

From the above analyzed data, and despite some controversial results which demand further clarification, the following conclusions are proposed. (a) Melatonin seems to promote bone formation and prevent bone resorption via several mechanisms which include the increase in the osteoblastic activity and differentiation, as well as the reduction in osteoclastic differentiation and activity, and by increasing osteoprotegerin expression and scavenging the free radicals responsible of bone resorption. (b) Melatonin may be an etiologic factor in the postmenopausal osteoporosis, and a therapeutic tool for this pathology, as an adjuvant with conventional treatments such as the administration of estrogens. (c) The recent data concerning the association of melatonin and AIS point toward their possible usefulness as both a diagnostic and therapeutic tool. (d) The experimental evidence on animal models suggests the value of clinical trials to assess the therapeutic possibilities of melatonin in bone diseases.
